# Advancing Treatment of Degenerative Eye Diseases at the Nanoscale

**DOI:** 10.1002/smmd.70050

**Published:** 2026-08-12

**Authors:** Li Yao Jin, Evashiniee G. P. Selvam, Qinsong Mei, Chi D. Luu, Yeannie H. Y. Yap, Daniel B. L. Teh

**Affiliations:** ^1^ Faculty of Science National University of Singapore Singapore; ^2^ Department of Ophthalmology Yong Loo Lin School of Medicine National University of Singapore Singapore; ^3^ Neurobiology Program Life Sciences Institute National University of Singapore Singapore; ^4^ Division of Applied Biomedical Science and Biotechnology School of Health Sciences IMU University Kuala Lumpur Malaysia; ^5^ Department of Medical Biochemistry and Molecular Biology School of Medicine Jinan University Guangzhou China; ^6^ Centre for Eye Research Australia Royal Victorian Eye and Ear Hospital Melbourne Victoria Australia; ^7^ Ophthalmology Department of Surgery University of Melbourne Melbourne Victoria Australia; ^8^ Department of Anatomy Yong Loo Lin School of Medicine National University of Singapore Singapore; ^9^ Department of Biomedical Engineering College of Design & Engineering National University of Singapore Singapore

**Keywords:** age‐related macular degeneration, aging, diabetic retinopathy, glaucoma, nanotechnology, ophthalmology

## Abstract

Degenerative ocular diseases are characterized by a convergence of molecular perturbations, including oxidative stress, chronic inflammation, failure of proteostasis, mitochondrial dysfunction, abnormal angiogenic signaling, and biomechanical remodeling. The eye's unique architecture, sealed by the blood‐retina barrier, presents formidable anatomical and cellular topological challenges to achieving long‐term curative therapeutics. Nanotechnology offers an advantage in potential therapy: by utilizing carriers in the nanometer size range and leveraging on appropriate surface modifications, it can effectively overcome these physiological barriers for enhanced, controlled, and targeted treatment. Clinical and preclinical systems such as antioxidant nanoparticles, sustained release implants, anti‐angiogenic nanoplatforms, multifunctional nanocomposites, and regenerative scaffolds illustrate how nanotechnology can overcome limitations in bioavailability, tissue penetration, and pathway specificity in degenerative ocular diseases. Diagnostic advances that include plasmonic contrast agents, quantum dots, graphene biosensors, smart contact lenses, and photothermal neuromodulators further demonstrate continuous molecular surveillance and responsive intervention. Emerging materials such as DNA origami and magnetoelectric nanosystems expand this capability by enabling direct modulation of transcriptional, metabolic, and neuroelectrical pathways. Together, these developments position nanotechnology as a powerful strategy for addressing the molecular origins of ocular degeneration while also highlighting ongoing challenges in long term safety, device stability, manufacturing reproducibility, and regulatory standardization.

## Highlights

- This review summarizes the molecular mechanisms underlying major degenerative ocular diseases and discusses how nanotechnology can overcome ocular biological barriers to improve targeted and sustained therapeutic delivery.
- This review highlights current clinical and preclinical nanomedicine strategies, including nanozymes, anti‐VEGF nanoplatforms, sustained‐release systems, regenerative nanoscaffolds, and multifunctional nanoparticles for ophthalmic applications.
- This review discusses emerging nanoscale diagnostic and monitoring technologies such as biosensors, smart contact lenses, wearable nanosystems, and stimuli‐responsive nanocarriers for precision ophthalmology.
- This review outlines key translational challenges in ocular nanomedicine, including biosafety, biodegradation, manufacturing reproducibility, and regulatory considerations, while prospecting future integration with AI‐driven and personalized therapeutic systems.

## Graphical abstract

Nanotherapeutics possess the potential to overcome longstanding bottlenecks in treating degenerative ophthalmic diseases. With a highly tunable surface chemistry, nanotechnology platforms can be customized to navigate specific anatomical barriers in the eye and target pathways of diseases.

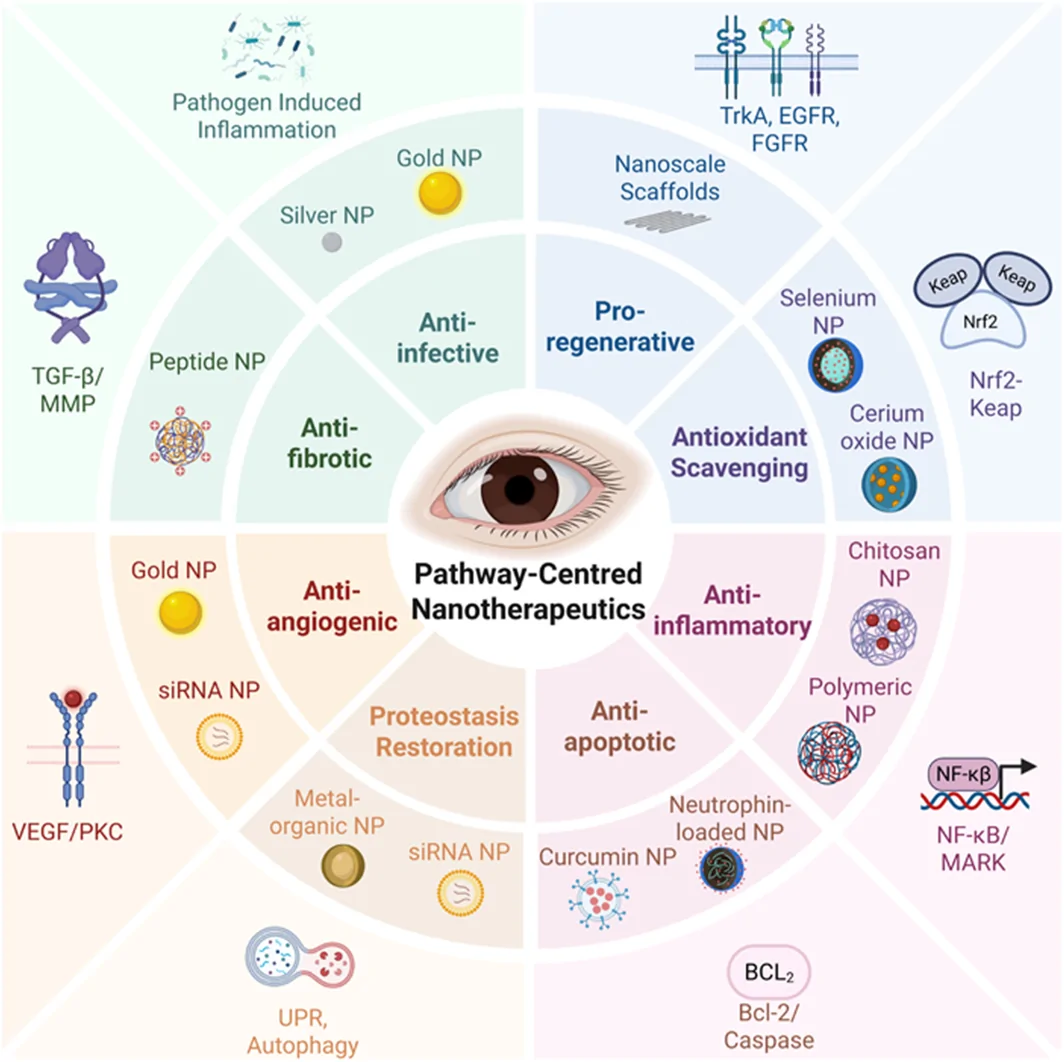

## 1 Introduction

Progressive ocular diseases involving degeneration of the retina and optic nerve, chronic inflammation, and age‐related changes such as glaucoma, age‐related macular degeneration (AMD), diabetic retinopathy (DR), cataract and degenerative and fibrotic corneal disorders can progressively compromise visual function [1, 2, 3, 4, 5, 6, 7, 8, 9, 10, 11] (Table 1). These conditions are often driven by overlapping pathological processes, including oxidative stress‐induced damage to mitochondrial membranes, lysosomal dysfunction, and disruption of intracellular protein quality‐control systems [12, 13, 14, 15, 16]. Because many of these processes occur at nanoscale biological interfaces, they present significant challenges for conventional therapies but may be amenable to modulation through nanotechnology‐based interventions.

**TABLE 1:** Major ocular diseases and their underlying pathological and molecular mechanisms.

Ocular disease	Key pathological mechanisms	Molecular mechanisms involved	Unique characteristics	References
Glaucoma	Retinal ganglion cell (RGC) death Optic nerve degeneration Elevated intraocular pressure (IOP)	Oxidative stress Neuroinflammation Mitochondrial malfunction Excitotoxicity Extracellular matrix (ECM) remodeling TGF‒β signaling BDNF/TrkB dysregulation Defective autophagy Endoplasmic reticulum (ER) stress	Chronic neuroinflammation via glial activation Resistance to IOP‒lowering therapy due to multifactorial pathology	[8, 10, 11]
Age‒related macular degeneration (AMD)	Degeneration of photoreceptors and retinal pigment epithelium (RPE) Drusen deposits between Bruch's membrane and RPE	Oxidative stress Chronic low‒grade inflammation Dysregulated complement activation Mitochondrial dysfunction Defective mitophagy ER stress	Genetic predisposition (complement factor H) Anti‒VEGF therapy only slows progression without reversing damage	[74, 75, 77]
Diabetic retinopathy (DR)	Microvascular damage Pre‒vascular neurodegeneration Glial remodeling	Hyperglycaemia‒induced VEGF upregulation • PKC activation AGE accumulation Oxidative stress Inflammatory cascade activation	Inner retinal dysfunction precedes vascular damage Early neurodegeneration requires neuroprotective intervention	[94, 101, 102]
Cataract	Lens opacification due to protein aggregation	Oxidative stress‒induced crystallin modification Disturbed calcium homeostasis Reduced antioxidant defences	Surgically correctable Lack of effective non‒surgical prophylactic treatments	[5, 12, 109]
Degenerative and fibrotic corneal disorders	Impaired corneal clarity from epithelial, stromal, or endothelial damage	ECM turnover dysregulation Cytokine imbalance Oxidative stress	Corneal transparency depends on highly organized stromal architecture Poor regenerative capacity of corneal endothelial cells	[4, 61]

Nanomaterials can interact with biological interfaces involved in disease progression and may facilitate targeted delivery of therapeutic agents to these sites [17, 18]. Nanotechnology is intrinsically at the nanometer scale with robustness for surface modification for the integration of functional or therapeutic agents [18, 19]. Beyond biochemical selectivity, the eye's biological barriers also impose significant cellular and topological constraints. For instance, the corneal epithelium presents tightly packed cells connected by tight junctions with nanometer‐scale paracellular gaps, while the stromal matrix consists of densely aligned collagen fibrils that restrict the diffusion of large molecules. Similarly, the blood‐retinal barrier is formed by highly polarized endothelial and retinal pigment epithelial cells that tightly regulate molecular transport. These structural constraints create a complex topological landscape that limits the penetration and distribution of conventional therapeutics [5, 20].

Nanocarrier systems can address these constraints through topological matching, whereby the physical form of nanomaterials, such as their size, geometry, surface topology, and mechanical flexibility, is tailored to navigate the spatial and structural features of ocular tissues [2, 5, 18]. Nanoparticles below specific size thresholds may diffuse through extracellular matrix pores [21, 22]. Surface modifications, including hydrophilic coatings or charge modulation, may further improve interactions with mucins and cellular membranes, enhancing tissue retention and transcellular uptake [23, 24]. In addition, mechanically deformable nanocarriers such as lipid‐based vesicles or polymeric micelles may adapt their morphology to pass through confined biological spaces [19, 25].

Nanotechnology offers unique physiochemical properties such as increased surface area, quantum effects, and enhanced biological interactions that enable precise control over chemical reactions and biological interfaces [17, 26, 27, 28, 29]. These properties form the foundation of nanomedicine, an interdisciplinary field that integrates nanotechnology, pharmacology, and biomedicine to revolutionize drug delivery and diagnostics [30, 31, 32]. Traditional small molecules and biologics may encounter difficulty in achieving sustained intraocular concentrations or in penetrating layered ocular barriers such as the corneal epithelium, retina, and blood‐retinal interface [4, 5]. In contrast, engineered nanoparticles can be designed to deliver therapeutic agents more efficiently to disease‐relevant molecular pathways [33]. In experimental systems, nanocarriers can be engineered to localize within specific organelles such as mitochondria, lysosomes, endoplasmic reticulum or targeted cells, enabling more localized delivery of therapeutic agents [34, 35, 36, 37, 38]. Surface functionalization enables selective interaction with retinal neurons, glia, vascular endothelium, or corneal epithelium, allowing nanoparticles to reach relevant cellular populations effectively [39, 40]. Stimuli‐responsive designs further permit on‐site release in response to oxidative stress, pH changes, enzymatic activity, or cytokine elevations, which are hallmark features of early ocular pathology [37, 41, 42].

**FIGURE 1:**
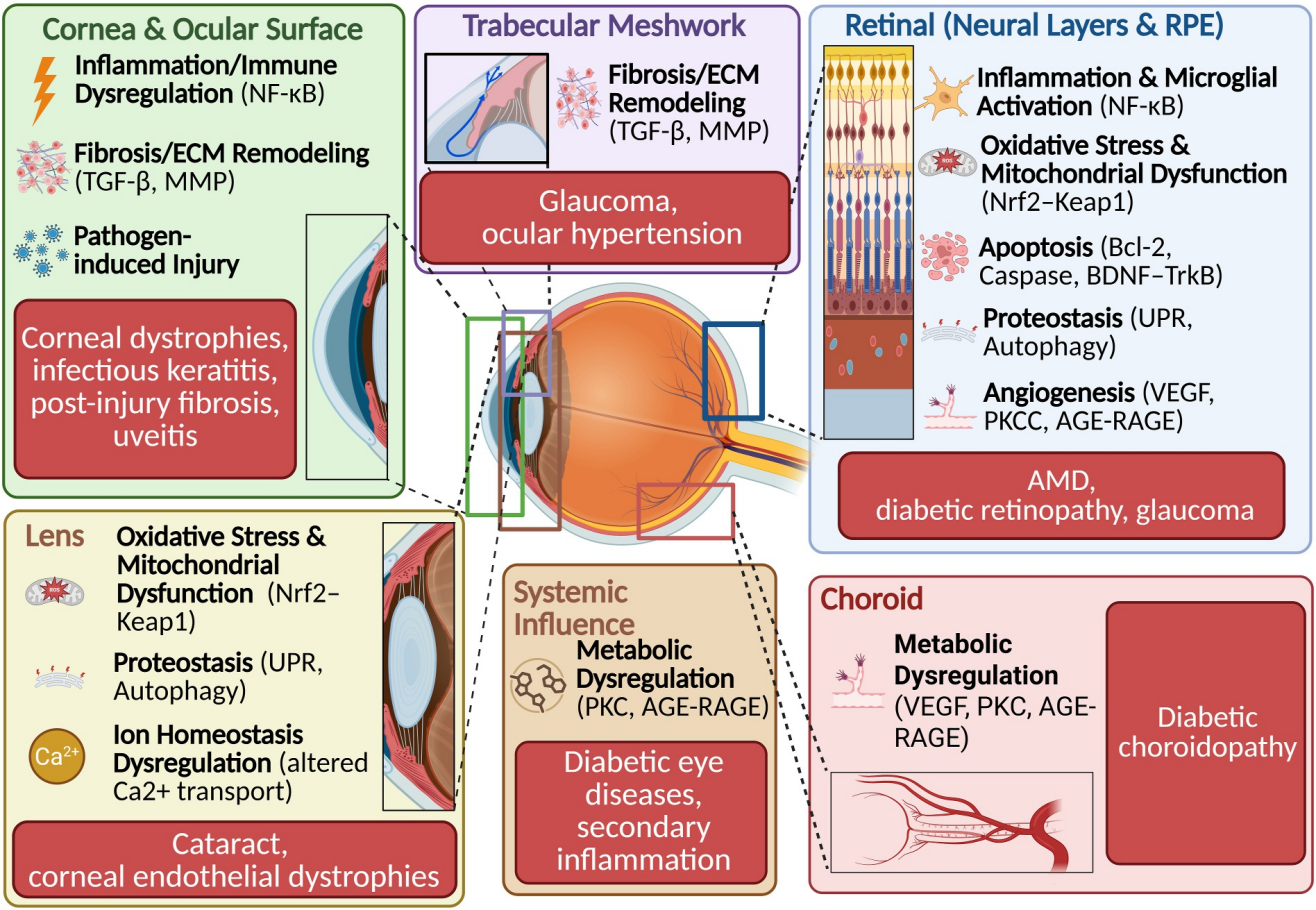
Anatomical mapping of molecular pathways underlying major ocular diseases. Common molecular mechanisms such as oxidative stress, inflammation, proteostatic failure, angiogenic activation, and fibrotic remodeling occur across distinct ocular compartments but manifest as different clinical entities depending on tissue context. Created with BioRender.com.

**FIGURE 2:**
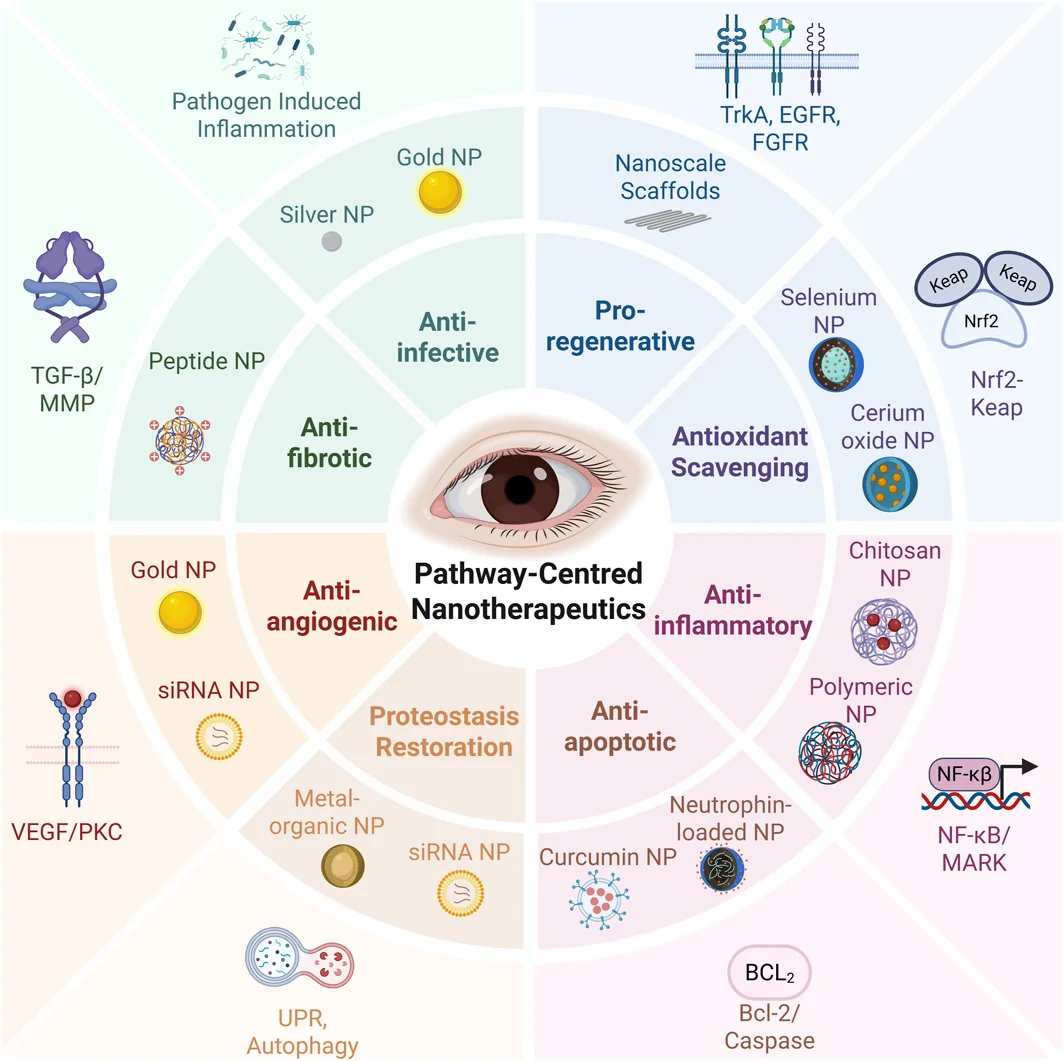
Schematic representation of nanotherapeutics targeting selected key molecular pathways in ophthalmology. Created with BioRender.com.

Furthermore, nanomedicine can support targeted regulation of key signaling axes such as Nuclear factor erythroid 2‐related factor 2 (Nrf2)‐Kelch‐like ECH‐associated protein 1 (Keap1), nuclear factor kappa‐light‐chain‐enhancer of activated B cells (NF‐κB), mitogen‐activated protein kinase (MAPK), vascular endothelial growth factor (VEGF)‐VEGF receptor (VEGFR), complement activation, EGFR signaling and endoplasmic reticulum (ER) stress responses, apoptotic pathways (Figure 1) [34, 35, 36, 37, 38, 43, 44, 45, 46, 47]. Nanotechnology encompasses engineered nanosystems and nanoparticles derived from a range of organic (lipid‐ and polymer‐based), inorganic (metallic, silica), and hybrid (organic‐inorganic composite) materials at the sub‐100 nm scale [17, 26, 27, 33, 37, 48, 49]. Within this operational boundary, ocular nanotechnologies can be categorized by their primary functional roles, including drug carriers (liposomes, polymeric nanoparticles), nanozymes (catalytic nanomaterials that modulate oxidative or inflammatory pathways), nanosensors (optical, electrochemical, or plasmonic platforms for molecular detection), and bioelectronic or magnetoelectric nanodevices designed to interface with neural or retinal tissue [17, 20, 39, 50, 51]. These nanoscale platforms can be rationally engineered to interface directly with surviving retinal circuitry bypassing retinal and optic nerve degeneration (e.g., inherited retinal diseases, age‐related macular degeneration, diabetic retinopathy, glaucoma), enabling vision restoration and even perception beyond the normal human visual range (e.g., near‐infrared) (Figure 2) [52, 53, 54]. This review evaluates ocular nanomedicine applications, translational challenges, and innovations such as smart nanocarriers, highlighting nanotechnology's transformative potential in ophthalmology.

## 2 Ocular Diseases and Nanotherapeutic Strategies

### 2.1 Glaucoma

**FIGURE 3:**
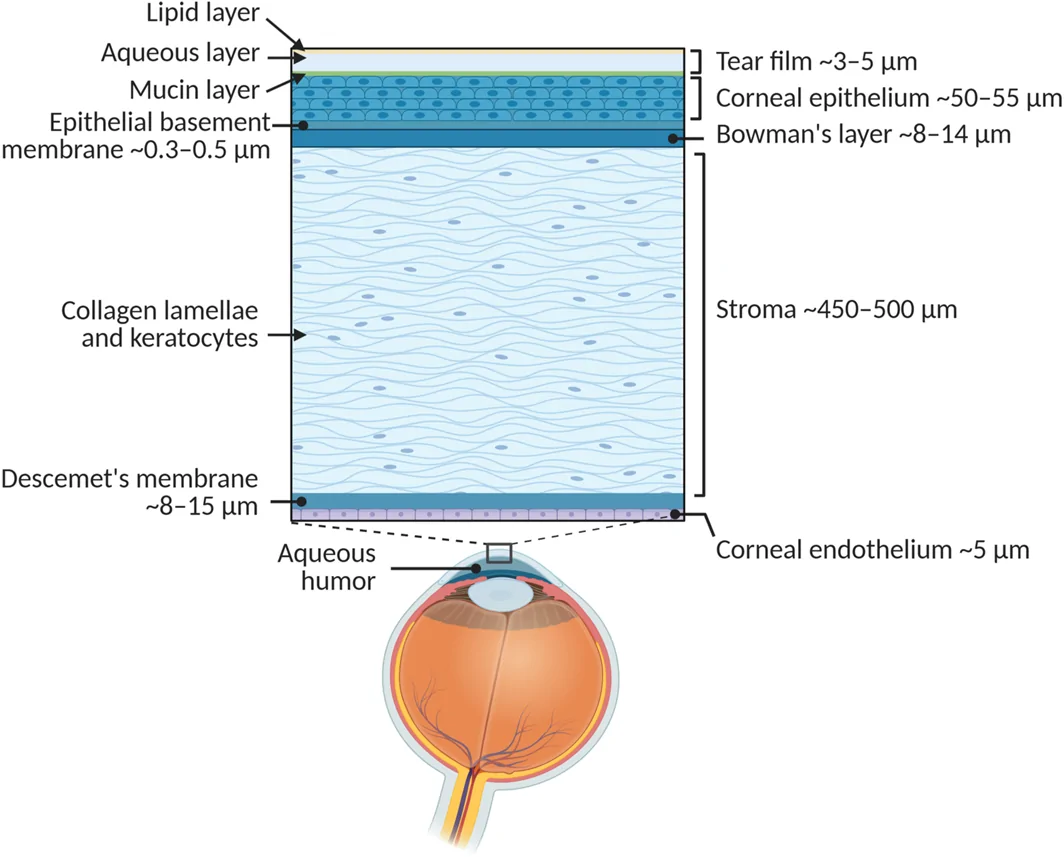
Schematic illustration of corneal structure. Adapted under terms of the CC‐BY license [61]. Copyright 2025, The Authors, published by Elsevier.

Current global estimates indicate that approximately 80 million people worldwide are to have glaucoma. This number is projected to rise significantly to over 111 million by 2040, driven primarily by an aging global population and the increasing prevalence of myopia [55]. Aging is a major risk factor for glaucoma, a progressive optic neuropathy characterized by irreversible loss of retinal ganglion cells and their axons, often, but not always associated with elevated intraocular pressure (IOP). Glaucoma is often asymptomatic in its early stages before progressing to irreversible visual loss [56]. Various contributory factors include accumulation of reactive oxidative species (ROS), inflammation remodeling and senescence of the trabecular meshwork, which results in its dysfunction, thus elevating the IOP [57, 58]. Although topical beta‐blockers, prostaglandin analogs, and carbonic anhydrase inhibitors effectively lower IOP, treatment outcomes are often inconsistent, primarily due to poor patient adherence, with limited corneal permeability as an additional challenge [59, 60]. The cornea functions as a selective barrier with an alternating lipophilic‐hydrophilic structure: a superficial lipophilic epithelium, a thick hydrophilic stroma, and an inner lipophilic endothelium. The multilayered architecture of the cornea presents a major barrier to topical ocular drug delivery, limiting transcorneal penetration and resulting in low bioavailability (< 5%) at the target site, necessitating specialized formulations such as penetration enhancers or nanoparticle‐based systems (Figure 3) [61, 62]. Drug delivery via topical eye drops is further limited by low ocular bioavailability due to rapid precorneal clearance, including tear turnover, blinking, and nasolacrimal drainage, which reduce corneal contact time and promote systemic absorption [63, 64]. For instance, prostaglandin analogs are relatively lipophilic, whereas non‐selective *β*—blockers such as timolol are comparatively hydrophilic, resulting in distinct permeation characteristics through the lipophilic corneal epithelium and hydrophilic stroma [47, 63]. Ideally, encapsulation within nanomicelles, which are self‐assembled spherical amphiphilic copolymers containing both hydrophobic and hydrophilic regions, can facilitate drug transport across the amphipathic cornea, thereby enhancing ocular penetration [65]. Similarly, polymeric nanoparticles derived naturally or synthetically may also be used to encapsulate the hydrophobic or hydrophilic drugs for ocular eye drop delivery [61].

For patients who do not respond to pharmacological therapy, surgical implantation may be required to control IOP. However, postoperative inflammatory responses and subconjunctival fibrosis remain the principal causes of long‐term implant failure by obstructing aqueous humor outflow [66]. Coatings such as poly(styrene‐b‐isobutylene‐b‐styrene) reduce fibrosis and prolong the therapeutic value of the implant. Sustained‐release implants and bimatoprost (prostaglandin analogues) inserts have the potential to maintain therapeutic intraocular drug concentrations over extended periods, although long‐term implant biocompatibility, safety, and validation of true neuroprotection remain unresolved [33, 67, 68, 69].

Although these systems are not strictly nanoscale technologies, clinically approved sustained‐release implants serve as important benchmarks for controlled intraocular drug delivery, providing a translational reference for emerging nanotechnology‐based therapeutic strategies. The bimatoprost intracameral biodegradable implant, Durysta, represents a polymer‐based depot placed in the anterior chamber that provides long‐acting prostaglandin analogue delivery in routine practice [69]. In the phase IV ARGOS 18 month prospective observational study enrolling 217 patients and 341 eyes, 88.6% of primary eyes required no new additional intraocular pressure lowering treatment through month 6%, and 77.7% still required none through month 18, alongside mean intraocular pressure reductions of about 1.0–2.0 mm Hg and a reduction in topical medication burden from 1.8 at baseline to 0.9 at month 12 and 1.0 at month 18 [69].

Another sustained delivery platform used clinically as a comparator for emerging nanotechnology strategies is the OTX‐TP (travoprost punctum plug) system, which uses poly(lactic acid) microspheres embedded within a bioresorbable hydrogel plug placed in the canaliculus to provide controlled release without daily drops [70]. In an initial feasibility, prospective, single arm study in an Asian population involving 17 subjects and 26 eyes, intraocular pressure reduction from baseline at day 10 was 6.2 mm Hg at 8 a.m., 5.4 mm Hg at 10 a.m., and 7.5 mm Hg at 4 p.m., corresponding to about 21%–28% reduction, while by day 30, plug retention declined to 42% and overall intraocular pressure reduction decreased to about 16% [70]. Long‐term safety data from post‐marketing surveillance will continue to clarify implant durability and chronic tissue responses.

Although no nanoparticle‐based glaucoma nanomedicine has yet achieved the same level of routine clinical adoption as approved sustained‐release implants, several nanoformulated topical therapies have entered early clinical evaluation. Dorzolamide *γ*‐cyclodextrin nanoparticle eye drops and dorzolamide‐loaded nanoliposome formulations have shown promising IOP‐lowering effects in small human studies, with the potential to reduce dosing frequency while maintaining acceptable tolerability [71, 72]. However, these platforms remain investigational and require larger, longer‐term clinical trials before they can be considered as clinically established glaucoma nanomedicines. In parallel, contact lens‐based drug delivery remains at the preclinical ocular stage, with registered preclinical and in vivo studies performed to assess the safety, tolerability, comfort, and feasibility of intraocular pressure lowering using a latanoprost eluting contact lens system, which aims to pair sustained release with passive wear‐based adherence [73].

### 2.2 Age‐Related Macular Degeneration (AMD)

Age‐related macular degeneration (AMD) is a chronic, multifactorial retinal disorder characterized by oxidative stress‐ and inflammation‐driven accumulation of lipid‐ and amyloid‐rich drusen beneath the retinal pigment epithelium (RPE), ultimately leading to retinal degeneration [74, 75, 76, 77, 78, 79] Although biological aging is the biggest risk factor of AMD, its risk is accelerated by smoking, obesity, and hypertension [77, 78]. Most patients initially present with early or intermediate AMD, characterized by drusen and pigmentary abnormalities, while a subset progresses to advanced disease as either neovascular (wet) AMD, marked by choroidal neovascularization (CNV), leakage, and fibrosis, or geographic atrophy (advanced non‐neovascular/dry AMD), characterized by progressive loss of the RPE, photoreceptors, and choriocapillaris, both of which result in irreversible central vision impairment [74, 75, 76, 77, 78, 79, 80, 81]. Current management remains limited: AREDS2 antioxidant supplementation reduces progression risk in intermediate AMD by approximately 25%, complement inhibitors such as pegcetacoplan and avacincaptad pegol slow geographic atrophy lesion growth, and anti‐VEGF intravitreal injections remain the standard of care for neovascular AMD, although none of these approaches halt or reverse disease progression completely [82, 83]. Subthreshold nanosecond laser has likewise demonstrated no significant overall benefit in delaying progression to late AMD in randomized clinical trials, although post hoc analyses suggest a potential benefit in selected patients without coexistent reticular pseudodrusen, warranting further investigation [84, 85].

Visudyne (verteporfin for injection) is one of the earliest clinically approved nanomedicine platforms for AMD. Formulated as a liposomal verteporfin nanomedicine for photodynamic therapy (PDT), it is clinically approved for the treatment of predominantly classic subfoveal CNV secondary to neovascular AMD. Following intravenous administration, verteporfin preferentially accumulates within CNV lesions and is activated by non‐thermal laser light, generating ROS that selectively occlude pathological neovasculature while minimizing damage to surrounding retinal tissues [86, 87, 88]. In the pivotal Treatment of AMD with Photodynamic Therapy (TAP) trials, verteporfin significantly reduced the risk of moderate and severe vision loss compared with placebo, with 61.2% of treated eyes losing fewer than 15 ETDRS letters at 12 months compared with 46.4% in the placebo group (*p* < 0.001; 86). These visual benefits were maintained through the 24‐month follow‐up [87], while the subsequent 3‐year open‐label extension demonstrated sustained long‐term safety without new treatment‐related safety concerns [87, 88]. Consistent with these findings, the Verteporfin in AMD (VAM) expanded‐access study involving 4435 patients across 222 centers confirmed a favorable real‐world safety profile, with treatment‐related adverse events reported in only 6.8% of patients, acute severe visual acuity loss occurring in 0.6%, and photosensitivity reactions in only 0.05% of patients [89]. Visudyne therefore represents a successful clinical translation of a liposomal nanomedicine, demonstrating the long‐term feasibility of nanocarrier‐based therapeutics in ophthalmology.

In contrast, most newer AMD nanoplatforms remain at the preclinical stage [90, 91, 92, 93] Representative preclinical platforms include biomimetic high‐density lipoprotein (HDL) nanoparticles for rapamycin delivery, astragaloside‐IV‐loaded lipid nanocapsules, atorvastatin‐loaded solid lipid nanoparticles, and DGR peptide‐functionalized nanoparticles co‐delivering miR‐150 and quercetin [90, 91, 92, 93]. These platforms aim to improve retinal drug delivery, prolong intraocular retention, and simultaneously target oxidative stress, inflammation, angiogenesis, and metabolic dysregulation in AMD. However, unlike Visudyne, they remain at the preclinical stage, underscoring the substantial translational gap between experimental nanomedicine and clinical application.

### 2.3 Diabetic Retinopathy (DR)

DR is a progressive retinal disorder caused by chronic hyperglycaemia and is a leading cause of vision loss among working‐age adults worldwide [6, 7, 13, 94, 95]. Prolonged exposure to high blood glucose levels results in biochemical and structural changes in the retinal microvasculature, leading to endothelial cell damage, pericyte loss, basement membrane thickening, and increased vascular permeability [94]. Early disease presents as non‐proliferative diabetic retinopathy (NPDR), characterized by microaneurysms, dot and blot hemorrhages, hard exudates, and subtle capillary nonperfusion. With disease progression, ischemia and hypoxia worsen, inducing pathological retinal neovascularization that defines proliferative diabetic retinopathy (PDR; [94, 96]). These fragile new vessels bleed into the vitreous and form fibrous scar tissue, which can cause tractional retinal detachment and severe visual loss [96]. In addition to vascular pathology, diabetic retinopathy involves neuroglial dysfunction and chronic low‐grade inflammation, contributing to neuronal death and disruption of the neurovascular unit [6, 7].

Current treatments for diabetic retinopathy focus on managing the late vascular and edema manifestations rather than preventing the underlying microvascular and neurodegenerative changes [94, 97]. For vision‐threatening diabetic macular edema, repeated intravitreal injections of anti‐vascular endothelial growth factor (anti‐VEGF) agents are the standard of care [98]. These treatments aim to reduce vascular leakage and central macular thickening. Frequent dosing is often required because the therapeutic effect of each injection is transient, while potential systemic adverse effects remain an ongoing safety consideration [99]. For proliferative diabetic retinopathy, panretinal laser photocoagulation is used to reduce ischemia‐driven neovascularization, but it is an ablative procedure that can result in peripheral visual field loss and irreversible retinal tissue damage [100]. Pars plana vitrectomy surgery is indicated for non‐clearing vitreous hemorrhage and tractional retinal detachment, but is invasive and associated with complications such as recurrent hemorrhage and retinal breaks [94, 101, 102]. Collectively, these treatments are effective for managing advanced disease but do not halt the fundamental metabolic stress, neurovascular dysfunction, and chronic inflammation that drive diabetic retinopathy progression. Despite advances in current therapies, many patients continue to experience progressive visual impairment or suboptimal visual outcomes, while frequent clinic visits and repeated invasive procedures impose a substantial treatment burden [103]. Consequently, recent reviews have highlighted growing interest in nanotechnology‐based strategies that aim to improve retinal drug delivery and enable more sustained therapeutic intervention in diabetic retinopathy [96, 101, 104].

One such example is the PEDF34 peptide encapsulated within biodegradable polymeric nanoparticles and evaluated in experimental models of ischemic and diabetic retinopathy [105]. This nanocarrier platform remains at the preclinical stage, with therapeutic efficacy demonstrated primarily in animal models rather than human clinical trials. According to Qu et al. (2022), in an oxygen‐induced retinopathy (OIR) model of ischemia‐driven retinal neovascularization, which shares mechanistic features with proliferative diabetic retinopathy, intravitreal administration of PEDF34 NP significantly suppressed inflammatory and angiogenic signaling at the molecular level [105, 106]. Western blot analysis demonstrated that retinal VEGF expression was reduced by 27% following PEDF34 NP treatment compared with control nanoparticle injection (*p* < 0.05), while expression of intercellular adhesion molecule 1 (ICAM 1), a key mediator of leukostasis and blood retinal barrier breakdown, was reduced by 31% (*p* < 0.01). These reductions were observed on postnatal day 18, a time point associated with active ischemia‐driven neovascular signaling. In parallel, PEDF34 NP treatment significantly decreased pathological neovascularization and vascular leakage, with therapeutic effects persisting for up to four weeks following a single intravitreal injection, whereas free peptide or control nanoparticles produced only transient or negligible effects [105]. Unlike standard anti‐VEGF biologics that require frequent reinjection to maintain efficacy, this prolonged intraocular activity highlights a core advantage of nanoparticle‐mediated delivery [107]. Importantly, PEDF34 NP modulated multiple pathological axes, including angiogenic, inflammatory, and fibrotic signaling pathways, supporting a broader disease‐modifying profile than single target therapies [105]. Such multi‐pathway engagement is particularly relevant given that diabetic retinopathy progression is driven not only by angiogenesis but also by chronic inflammation, blood retinal barrier dysfunction, and neuroglial injury [6, 94, 102]. Though cytotoxicity associated with nanoparticle‐mediated small peptides was ruled out [105, 107], the long‐term metabolic fate of polymeric nanoparticles within retinal tissues, including potential accumulation in retinal pigment epithelial cells or microglial populations, remains incompletely characterized and represents an important consideration for future clinical translation.

A complementary nanotechnology approach aims to overcome the invasiveness of intravitreal injection by enabling posterior segment drug delivery through topical administration, as demonstrated by Shoval et al. (2019). Anti‐VEGF aptamer functionalized carbon dots were shown to act as effective carriers across the corneal barrier, achieving therapeutic intraocular drug levels following eye drop application. These systems remain at the preclinical stage and have primarily been evaluated in experimental models rather than clinical studies. In preclinical rat models, topical administration of the carbon‐dot‐aptamer construct produced intraocular drug concentrations several‐fold higher than those achieved with free aptamer alone, demonstrating improved ocular penetration and bioavailability. Functionally, the system inhibited VEGF‐mediated angiogenesis with efficacy comparable to established anti‐VEGF biologics, suggesting potential utility for VEGF‐driven retinal vascular diseases such as diabetic retinopathy [108].

Although these findings remain preclinical, they illustrate how nanoscale engineering can markedly enhance ocular barrier penetration, enable real‐time pharmacokinetic monitoring, and achieve anti‐angiogenic activity comparable to injected biologics, highlighting capabilities that are not readily attainable with conventional topical or intravitreal formulations.

### 2.4 Cataract

Cataract development is strongly associated with oxidative stress, including ROS‐driven damage to lens proteins and antioxidant depletion, with emerging evidence implicating lipid peroxidation and ferroptosis‐related pathways in lens epithelial cell injury [109]. Conventional agents cannot reverse crystallin misfolding or reach therapeutic intra‐lenticular concentrations, making surgery the only definitive treatment [110]. Traditional small‐molecule antioxidant approaches, including low‐molecular‐weight antioxidants and glutathione‐related strategies, rely largely on direct redox reactions and may have limited durability compared with catalytic antioxidant systems or enzyme‐mimicking nanomaterials [111]. In contrast, nanozymes exhibit enzyme‐like catalytic activity that mimics endogenous antioxidant enzymes [111]. These nanomaterials can repeatedly decompose ROS through redox cycling mechanisms, enabling sustained catalytic ROS detoxification [112, 113]. For instance, cerium‐based nanoparticles undergo reversible Ce^3+^/Ce^4+^ redox transitions, allowing continuous ROS scavenging and mitigation of oxidative stress in experimental cataract models [114, 115]. Yang et al. demonstrated that CeCl_3_@mSiO_2_ nanoparticles reduced oxidative damage and delayed diabetic cataract progression, supporting the potential of catalytic nanomaterials to mitigate ROS‐driven lens damage. Beyond cerium systems, other nanozyme platforms, including manganese dioxide and selenium nanoparticles, have also been explored for catalytic ROS regulation and protection against crystallin oxidation and intracellular glutathione depletion [114, 115]. Importantly, the catalytic efficacy of these nanozymes can be tuned through nanoscale structural engineering, including particle size, surface defects, and heterostructure interfaces, which modulate electron transfer pathways and reactive oxygen species decomposition kinetics [116]. Yolk‐shell CeO_2_‐MnO_x_ heterostructures and Prussian blue@CeO_2_ core–shell nanozymes maintain regenerative catalytic activity for over 30 days in aqueous humor mimics, leveraging endogenous reductants [50]. Gene‐nanomedicine approaches, such as LNP‐mRNA encoding human lanosterol synthase, enable sustained expression of human lanosterol synthase in the lens and improve cataract severity in rat models, supporting the feasibility of reversing early cataract pathology [117]. Overall, nano‐enabled cataract therapies, including antioxidant nanozymes and gene delivery, remain preclinical but offer a promising foundation for non‐surgical cataract management.

### 2.5 Corneal Disorders

Progressive corneal disorders, including stromal fibrosis and Fuchs endothelial corneal dystrophy, are characterized by distinct pathological mechanisms. Stromal fibrosis arises from persistent TGF‐β‐driven myofibroblast activation and extracellular matrix remodeling following dysregulated wound healing, whereas Fuchs endothelial corneal dystrophy involves progressive endothelial cell loss and dysfunction, resulting in impaired endothelial pump function and corneal edema [118, 119]. Collectively, corneal diseases remain a leading cause of blindness worldwide, exacerbated by a severe global shortage of donor corneas for transplantation, particularly in low‐ and middle‐income regions where demand far exceeds supply and access to keratoplasty remains limited [120, 121]. These pathologies are further compounded by the eye's multilayered anatomical barriers, which limit drug penetration and create low bioavailability for conventional drops, gels, or systemic therapy [18, 20]. As reviews highlight, conventional ocular therapies often provide only temporary or disease‐specific benefit, while topical and injectable delivery remain limited by rapid clearance, low bioavailability, short residence time, and static and dynamic ocular barriers [4, 122, 123, 124, 125].

Recent nanomedicine strategies offer potential solutions to these mechanistic limitations through sustained and targeted delivery of therapeutics that modulate fibrosis, oxidative stress, and endothelial dysfunction [119, 122]. Although most studies remain at the preclinical stage and have primarily been evaluated in experimental models of corneal injury or fibrosis rather than naturally occurring degenerative disorders, they provide important proof‐of‐concept for mechanism‐directed therapy [122]. For example, pirfenidone‐loaded nanoparticles reduced TGF‐β‐mediated collagen I and *α*‐smooth muscle actin expression while attenuating stromal scarring after alkali injury, whereas gold nanoparticle‐mediated BMP7 gene delivery suppressed corneal haze, myofibroblast differentiation, and extracellular matrix remodeling in vivo [126, 127]. Similarly, polyethyleneimine‐based nanoparticles delivering TGF‐β1 siRNA have been investigated to inhibit profibrotic signaling and stromal fibrosis [128]. For corneal endothelial disorders, lipid nanoparticles have demonstrated efficient nucleic acid delivery to endothelial cells, supporting the future development of gene‐ and RNA‐based therapies for degenerative conditions such as Fuchs endothelial corneal dystrophy, where oxidative stress, endothelial cell loss, and endothelial pump dysfunction remain key therapeutic targets [129, 130]. Collectively, these studies highlight the potential of nanomedicine to enable sustained, mechanism‐directed modulation of progressive corneal pathology, although further validation in disease‐specific models and clinical studies is required [119, 122].

Despite substantial progress, several challenges continue to limit the clinical translation of nanomedicine for corneal disorders. Achieving sustained drug retention, controlled release, long‐term stability of biologics and nucleic‐acid therapeutics, and reproducible large‐scale manufacturing while preserving optical transparency and biocompatibility remain key barriers to clinical implementation [119, 123]. Future efforts should focus on clinically scalable nanocarriers capable of simultaneously modulating fibrosis, oxidative stress, and endothelial dysfunction, together with rigorous evaluation of long‐term safety, biodegradability, and therapeutic efficacy in disease‐specific preclinical and clinical models [119, 123]. Continued advances in these areas may enable nanomedicine to address the underlying molecular mechanisms of progressive corneal disease and ultimately reduce reliance on donor corneal transplantation.

Beyond degenerative eye diseases, nanotherapeutics have also demonstrated broad applicability in other ocular conditions [131]. Ocular infections, including bacterial keratitis, fungal keratitis, and endophthalmitis, are major causes of vision loss worldwide [132, 133]. These infections disrupt ocular surface homeostasis, trigger inflammatory responses, and ultimately contribute to tissue damage and vision loss [132, 134].

Polymeric nanoparticles, liposomes, and hydrogel‐based carriers can enhance corneal residence time and improve drug bioavailability relative to conventional topical formulations [2, 20, 135]. Their therapeutic potential has been demonstrated in preclinical models of ocular infection, where nanoformulations of antimicrobial agents achieved improved corneal drug delivery and treatment efficacy compared with conventional formulations [135]. These findings illustrate the broader advantages of nanoscale drug delivery, including enhanced tissue targeting, prolonged drug retention, reduced dosing frequency, and improved therapeutic outcomes, which are equally applicable to degenerative eye diseases.

Table 2 summarizes representative nanotechnology‐based therapies discussed throughout this review. However, most nanocarrier systems discussed above remain at the preclinical or early translational stage, and their long‐term ocular pharmacokinetics, biodegradation pathways, and cumulative biosafety profiles will require careful evaluation before widespread clinical adoption.

**TABLE 2:** Representative nanomedicine platforms and selected clinical sustained‐delivery systems for ocular diseases.

Disease	Therapeutic platform	Drug/agent	Mechanistic target	Delivery system	Status/approval	Therapeutic focus	References
Glaucoma	Clinical sustained‐release implant (Durysta)	Bimatoprost (prostaglandin analogue)	FP receptor agonism → ↑ uveoscleral (± trabecular) outflow → IOP lowering	Intracameral biodegradable implant placed in the anterior chamber (polymer “depot”)	FDA‐approved for open‐angle glaucoma (OAG) and ocular hypertension (OHT)	Long‐acting IOP reduction, reduced topical medication burden; benchmark for sustained intraocular drug delivery	[69]
	Sustained‐release punctum plug (OTX‐TP)	Travoprost (prostaglandin analog)	FP receptor agonism → ↑ uveoscleral outflow → IOP lowering	Bioresorbable hydrogel punctum plug (canaliculus) with PLA microspheres for controlled release	Clinical feasibility study	Sustained topical prostaglandin delivery without daily eye drops; translational comparator for controlled ocular drug delivery	[70]
AMD	Liposomal photodynamic therapy (Visudyne)	Verteporfin	Light activation generates reactive oxygen species (ROS) → selective occlusion of choroidal neovascularization (CNV) with minimal damage to surrounding retina	Intravenous liposomal nanocarrier for photodynamic therapy	FDA‐approved for predominantly classic subfoveal CNV secondary to neovascular AMD	Targeted ablation of pathological CNV; first clinically translated ophthalmic nanomedicine	[86, 87, 88, 89]
DR	PEDF34 peptide‐loaded polymeric nanoparticles	PEDF34 peptide	PEDF‐mediated anti‐angiogenic and anti‐inflammatory signaling → downregulation of VEGF and ICAM‐1, reducing neovascularization and vascular leakage	Biodegradable polymeric nanoparticles	Preclinical	Sustained anti‐inflammatory, anti‐angiogenic and anti‐leakage therapy	[105, 106, 107]
	Carbon nanodots with anti‒VEGF	Anti‐VEGF aptamer	VEGF neutralization combined with enhanced transcorneal penetration for posterior segment delivery	Carbon‐dot nanocarrier administered as topical treatment	Preclinical	Non‐invasive retinal drug delivery with anti‐angiogenic activity and improved ocular bioavailability	[108]
Cataract	Cerium‐based antioxidant nanozymes	Cerium oxide nanoparticles (CeO_2_), CeCl_3_@mSiO_2_ nanoparticles	Ce^3+^/Ce^4+^ redox cycling nanozyme activity mimicking superoxide dismutase and catalase plus inhibition of glucose induced protein glycation and ferroptosis in lens epithelial cells → reduction of oxidative stress, protein aggregation and diabetic cataract progression	Cerium‐based nanozymes (including mesoporous silica‐supported formulations)	Preclinical	Sustained catalytic ROS detoxification to delay cataract progression	[111, 112, 113, 114, 115, 116]
Corneal disorders	Pirfenidone‐loaded nanoparticles	Pirfenidone	Inhibition of TGF‐β‐mediated profibrotic signaling → reduced collagen I and α‐SMA expression, attenuating stromal fibrosis and corneal scarring	Polymeric nanoparticles	Preclinical	Anti‐fibrotic therapy for corneal stromal fibrosis	[126]
	Gold nanoparticle mediated gene delivery	BMP7 plasmid DNA	BMP7 overexpression antagonizes TGF‐β‐driven myofibroblast differentiation and extracellular matrix remodeling	Gold nanoparticles	Preclinical	Gene therapy to reduce corneal haze and fibrosis	[127]
	siRNA nanotherapy	TGF‐β1 siRNA	Silencing of TGF‐β1 expression to inhibit profibrotic signaling and stromal fibrosis	Polyethyleneimine‐based nanoparticles	Preclinical	RNA interference for corneal fibrosis	[128]
	Lipid nanoparticle nucleic acid delivery	Nucleic acids (gene/RNA therapeutics)	Efficient delivery of nucleic acids to corneal endothelial cells targeting oxidative stress, endothelial dysfunction and cell loss	Lipid nanoparticles	Preclinical	Gene/RNA therapy for endothelial disorders including Fuchs endothelial corneal dystrophy	[129, 130]

*Note:* Durysta and OTX‐TP are included as clinically approved sustained‐release delivery systems that serve as translational benchmarks for emerging nanotechnology‐based ocular therapeutics. Similarly, most corneal disease platforms listed have been evaluated in experimental models that recapitulate key mechanisms of progressive corneal disease.

## 3 Nanotechnology‐Enabled Diagnostics and Precision Therapeutics

### 3.1 Nanotechnology for Ocular Diagnostics and Monitoring

New nanotechnology platforms are also advancing ocular diagnostics and monitoring through ultra‐sensitive and multifunctional detection capabilities [122, 136, 137, 138]. Nanostructured contrast agents such as PEGylated gold nanorods and nanodisks enhance optical coherence tomography and photoacoustic imaging through strong surface plasmon resonance (SPR) that amplifies optical absorption and acoustic pressure generation [138]. This SPR‐mediated contrast enhancement improves photoacoustic signal generation and detection sensitivity, enabling earlier visualization of microvascular alterations associated with oxidative stress, VEGF‐driven angiogenesis, and endothelial dysfunction [136, 138].

Semiconductor quantum dots provide bright, photostable probes for multiplexed imaging, facilitating the detection of early molecular changes such as oxidative stress and inflammation before overt structural and clinical manifestations of AMD and DR [139]. Although AMD is typically slow‐progressing and currently lacks disease‐modifying therapies at its earliest stages, identification of these subclinical changes facilitates improved risk stratification, closer disease monitoring, and timely enrollment into interventional trials [74, 77]. Importantly, as emerging therapeutic strategies targeting oxidative and complement‐mediated pathways are expected to be most effective prior to irreversible retinal damage, sensitive early‐stage molecular imaging may become increasingly clinically relevant.

Nanostructured biosensors extend molecular surveillance by detecting biochemical, mechanical, and redox perturbations that precede clinically observable disease [140]. Graphene field‐effect transistor aptamer sensors quantify tear cytokines at picomolar concentrations, enabling minimally invasive tracking of inflammatory mediators associated with NF kappa B activation, mitochondrial stress, and early glial reactivity [140]. Early identification of these molecular perturbations has been shown to improve disease stratification, guide the timing and intensity of anti‐inflammatory or antioxidant interventions, and enable earlier detection of patients at higher risk of progression to irreversible retinal damage [141]. Such biomarker‐guided monitoring is increasingly recognized as critical for optimizing treatment response and evaluating emerging therapies targeting early inflammatory and oxidative pathways [12, 141]. Importantly, longitudinal tear‐based biosensing offers a non‐invasive means to assess therapeutic efficacy in real time, enabling adaptive treatment strategies before structural retinal changes become irreversible.

**FIGURE 4:**
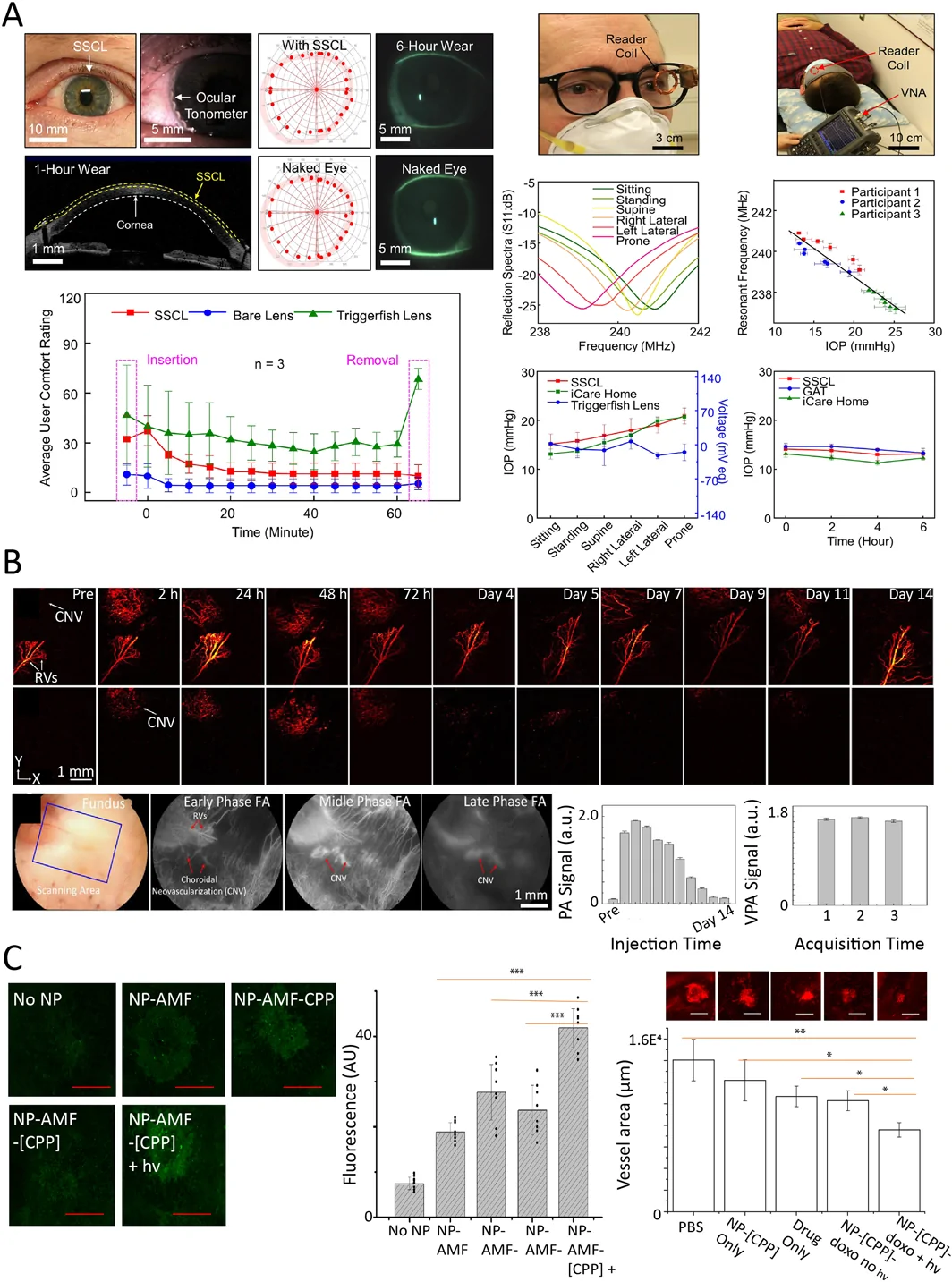
Representative wearable/implantable nanodevices and smart or stimuli‒responsive nanocarriers. (A) In‐clinic examinations of SSCL in human eyes, the visual field of the eye wearing the SSCL remained unchanged as compared to the naked eye; participants reported minimal discomfort; ambulatory IOP monitoring in human eyes. Adapted under terms of the CC‐BY license [142]. Copyright 2022, The Authors, published by Springer Nature. (B) PA detection of CNV in living rabbits using CGNP clusters‒RGD. Rabbit injected with CGNP clusters‐RGD exhibited significantly higher PA signal than pre‐injection, with steady in vivo photostability of CGNP clusters‐RGD. Adapted under terms of the CC‐BY license [137]. Copyright 2021, The Authors, published by Springer Nature. (C) Light‒triggered targeting of CNV in vivo; Treatment with NP‒[CPP]‒doxo in mouse CNV model. Representative isolectin GS IB4‐stained CNV images (the scale bar is 100 μm) and mean CNV lesion area from laser‐induced CNV mice treated with PBS, NP‐[CPP], doxo, NP‐[CPP]‐doxo, and NP‐[CPP]‐doxo with irradiation. Adapted under terms of the CC‐BY license [144]. Copyright 2019, The Authors, published by Springer Nature.

Wearable and implantable nanosystems complement biochemical sensors by capturing biomechanical and oxidative stress signals in vivo [51, 54, 137, 142, 143, 144]. Smart soft contact lenses have emerged as multifunctional wearable platforms capable of continuously monitoring both biomechanical and biochemical ocular biomarkers. In glaucoma, nanowire‐based intraocular pressure sensors provide continuous monitoring, linking fluctuations in ocular biomechanics to apoptosis and mechanotransduction‐dependent remodeling of the trabecular and retinal interface (Figure 4A) [142]. These capabilities have been further enhanced by next generation contact lenses integrating transparent silver and graphene nanowire networks, enabling high‐fidelity pressure monitoring with real‐time wireless communication [143]. Beyond glaucoma, smart contact lenses are also being explored for diabetes and diabetic retinopathy through the integration of nanosensors capable of continuously monitoring tear glucose and other tear biomarkers associated with metabolic dysfunction and retinal disease progression [145].

Implantable graphene and carbon nanotube sensors add complementary functionality by quantifying oxidative and electrochemical fluctuations within the anterior chamber [51]. These measurements provide a direct readout of Nrf2‐mediated antioxidant defense, redox instability, and inflammatory perturbations that drive early glaucoma progression [51]. Beyond sensing applications, photothermal neuromodulation systems such as intravitreally delivered plasmonic gold nanorods demonstrate how nanoscale photonic materials can interface directly with retinal circuitry [146].

### 3.2 Stimuli‐Responsive Nanoplatforms for Precision Therapeutics

Beyond diagnostic and monitoring applications, nanotechnology also enables externally controlled therapeutic intervention through stimuli‐responsive nanomaterials. Optically responsive nanosystems provide precise spatial and temporal control over therapeutic activation, enabling targeted modulation of pathological pathways while minimizing off‐target effects [137, 144]. In models of CNV, photoacoustic contrast nanoparticles and light‐activated nanocarriers facilitate localized near‐infrared (NIR)‐triggered drug release or photothermal therapy, suppressing VEGF and VEGFR2 signaling, limiting hypoxia‐induced angiogenic cascades, and attenuating subsequent vascular remodeling (Figure 4B,C) [137, 144].

Wearable therapeutic platforms have similarly expanded to smart contact lenses for retinal diseases. A wirelessly powered near‐infrared light‐emitting smart contact lens has recently been developed for diabetic retinopathy, integrating a flexible wireless power transfer system with an embedded micro‐light‐emitting diode (micro‐LED) capable of delivering repeated retinal photobiomodulation. In diabetic rabbit models, treatment with 120 μW NIR irradiation for 15 minutes, three times weekly over 8 weeks effectively prevented the development of diabetic retinopathy without detectable corneal or retinal toxicity, demonstrating the feasibility of non‐invasive wearable nanotechnology for retinal therapy [147].

Contact lens‐based therapeutic platforms are also being explored for infectious keratitis. Recent developments include multifunctional contact lenses incorporating antimicrobial nanocoatings that actively inhibit bacterial colonization while maintaining optical transparency and durability, as well as contact lens‐based sustained antibiotic delivery systems that achieve prolonged local drug release and enhanced corneal antibiotic exposure compared with conventional topical therapy [148, 149]. These advances further highlight the versatility of smart and multifunctional contact lens technologies as emerging platforms for precision ocular therapeutics, with potential translational relevance to degenerative eye diseases.

Despite these advances, several challenges remain before widespread clinical translation can be achieved. Activation thresholds for stimuli‐responsive systems can vary across ocular microenvironments, long‐term biocompatibility requires further study, and integration of sensing, processing, and therapeutic output in a stable ocular interface remains technically demanding [51, 54, 137, 142, 143, 144]. Continued refinement of pathway specificity, device stability, and biocompatibility will be essential for translating these nanotechnology‐enabled platforms into clinical practice.

Together, these diagnostic and therapeutic nanotechnology platforms illustrate the growing convergence of molecular sensing, physiological feedback, and precision intervention. Table 3 summarizes representative diagnostic and therapeutic nanosystems together with the principal molecular pathways they interrogate or modulate, including oxidative stress, angiogenic signaling, neurodegenerative cascades, and biomechanical stress responses.

**TABLE 3:** Representative wearable/implantable nanodevices and smart or stimuli—responsive nanocarriers with relevance to ocular nanomedicine and molecular pathway targeting.

Wearable/smart biosensors	Mechanism/trigger	Eye application/relevance	Targeted molecular pathway/mechanism	References
Smart soft contact lenses for 24‒hour IOP monitoring	Embedded nanowire sensors detect continuous intraocular pressure fluctuations	Enables responsive glaucoma management and early neuroprotection	Mechanotransduction‐induced RGC apoptosis (BCL‐2 downregulation and caspase activation)	[142]
Nanocomposite‒based ocular sensors	Graphene or CNT‒based biosensors measure biomechanical and electrochemical changes	Real‒time IOP and redox monitoring in glaucoma	Oxidative‒stress‐induced mitochondrial and apoptotic signaling (Nrf2‐Keap1; BCL‐2/Caspase)	[140]
Smart contact lenses with graphene nanowire IOP sensors and wireless circuits	Transparent Ag/graphene nanowire network senses IOP and supports wireless feedback	Combines sensing with a potential drug‒release interface for glaucoma	Oxidative‐stress‐linked redox and inflammatory signaling (Nrf2‐Keap1; NF‐κB axis)	[51, 143]
Plasmonic gold‐nanorod retinal photothermal prosthesis	Photothermal activation using patterned NIR laser (980 nm)	Intravitreal Thy1‒AuNRs bind retinal neurons and enable high‒resolution patterned stimulation of bipolar cells; evokes cortical responses even in blind mice	Activation of temperature‒sensitive TRPV channels (TRPV1/TRPV4); downstream Ca^2+^ signaling in bipolar cells; synaptic transmission to RGCs	[54]
Chain‐like gold nanoparticle clusters for multimodal OCT‐PA imaging	Surface plasmon resonance‐enhanced optical absorption and acoustic pressure generation	High‐sensitivity visualization of choroidal and retinal microvasculature; early detection of oxidative and angiogenic dysregulation	SPR‐enhanced amplification of optical absorption and acoustic signal generation, enabling high‐contrast detection of VEGF‐driven neovascular remodeling, endothelial oxidative stress, and microvascular instability.	[137]
Light‐activated NP‐CPP‐doxorubicin for CNV targeting	NIR‐triggered cleavage of photocages enabling site‐specific nanoparticle binding and drug release	Selective ablation of choroidal neovascular lesions with minimal off‐target toxicity	Targeted suppression of VEGF/VEGFR2 signaling and hypoxia‐induced angiogenic cascades via photo‐targeted nanoparticle accumulation, with localized cytotoxic modulation of proliferating endothelial cells upon NIR activation.	[144]

## 4 Emerging Nanotechnologies for Ocular Applications

**FIGURE 5:**
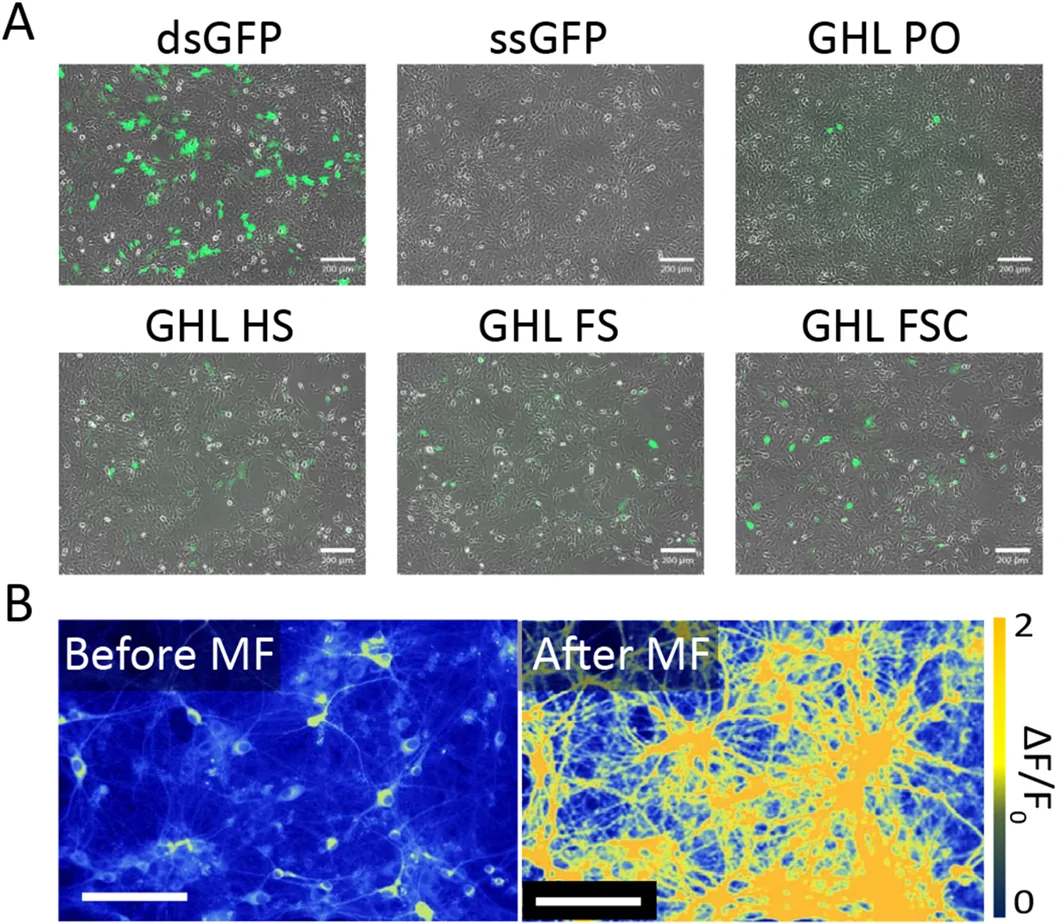
Representative imaging and molecular analyses illustrating the diverse functionalities of advanced nanomaterials in cellular systems. (A) Qualitative fluorescence imaging of GFP expression from various DNA nanoparticle architectures in mammalian cell culture. All constructs supported GFP expression above that of single‒stranded DNA (ssGFP). Scale bar 200 μm. Adapted with permission [150]. Copyright 2024, The Authors, published by Springer Nature. (B) Live‒cell calcium imaging of hippocampal neurons decorated with magnetoelectric nanoparticles (MENDs), showing relative GCaMP6s fluorescence change (ΔF/F_0_) before and after magnetic field stimulation (10 s, OMF 220 mT; AMF 1 kHz, 10 mT), indicating magnetically induced neuronal activation. Adapted under terms of the CC‐BY license [157]. Copyright 2025, The Authors, published by Springer Nature.

While ophthalmology has advanced rapidly in nano‒enabled drug delivery, many nanotechnologies pioneered in other biomedical domains remain underexplored for ocular applications. These systems currently provide non‐ocular conceptual evidence rather than direct ocular validation, extending beyond traditional carriers by providing molecular‐level precision and dynamic feedback control, complementing responsive nanocarriers and implantable platforms. Figure 5 illustrates representative imaging demonstrating how advanced nanomaterials engage distinct cellular pathways that can be translated toward ocular molecular therapy, including genetic modulation and neuronal activation [150, 151, 152, 153, 154, 155, 156, 157].

DNA origami nanostructures exemplify this next generation of precision materials [158]. Their programmable nucleic acid scaffolds allow nanoscale spatial control of gene or siRNA cargo [158]. Douglas et al. reported a DNA origami‐based nanorobot with a barrel‐like architecture designed for targeted payload presentation at the surface of specific cells. These nanorobots were engineered to carry single‐stranded DNA‐tagged nanoparticles or antibody fragments through sequence‐specific hybridization to complementary strands positioned within the internal cavity. The structures were sealed using aptamer‐based molecular logic gates that remained closed until recognizing defined receptors on target cell membranes. Upon receptor binding, the origami underwent a programmed conformational change that exposed the functional payload at the cell surface. Through this controlled interaction between the payload and cell surface receptors, the nanorobots enabled precise modulation of receptor‐mediated phosphorylation signaling in target cells [158]. This programmable and receptor‐specific control over payload exposure could enable highly precise ocular drug delivery, allowing therapeutic signaling to be modulated at defined retinal cell populations while minimizing off‐target effects and systemic exposure. Beyond enabling spatially precise drug delivery, DNA origami platforms offer a framework for regulating downstream intracellular events, with emerging applications in the selective modulation of transcriptional pathways (Figure 5A) [150, 151]. These findings suggest a possible route for targeting retinal degenerative mechanisms. These findings illustrate the programmable capabilities of DNA origami nanostructures for receptor‐specific molecular interactions. Although these systems have not yet been widely applied in ophthalmology, their modular architecture may provide a conceptual framework for future nanoscale delivery systems targeting retinal cells. However, significant challenges remain, including nuclease degradation, intravitreal stability, immune responses, and efficient penetration across ocular barriers.

Cell membrane‐coated nanoparticles, which use endogenous erythrocyte or leukocyte membranes for cloaking, similarly enhance intravitreal stability and immune evasion. This property is essential for sustained modulation of chronic inflammatory circuits, complement activation, and innate immune surveillance within the posterior segment [152, 153, 154].

Other emerging nanomaterials aim to modulate molecular pathways directly. Zn‐based multi‐active framework nanoparticles TSA‐CAN‐Zn, are shown to suppress advanced glycation end product (AGE) accumulation and lysosomal overload, modulating the AGE‐Receptor‐for‐Advanced‐Glycation‐End‐products (RAGE) metabolic axis to restore redox and proteostatic balance [155]. These data support the concept that optical or energy‐responsive nanomaterials can modulate stress‐responsive transcription factors at the molecular level.

Magnetoelectric nanomaterials extend this principle by converting external physical inputs into biologically relevant electrical potentials. Magnetoelectric nanoparticles have been shown to activate BCL‐2‐associated apoptotic signaling and caspase cascades under magnetic stimulation [156]. Magnetoelectric neuromodulators can also generate local electrical stimulation that promotes calcium influx and upregulates neurotrophic signaling pathways, including BDNF and TrkB expression [157]. Direct evidence of magnetically evoked neuronal activation has been demonstrated in hippocampal cultures treated with magnetoelectric nanoparticles, in which magnetic‐field exposure produced robust calcium transients (Figure 5B) [157]. Such bioelectronic nanointerfaces represent a growing class of nanosystems with potential applications in modulating retinal excitability through non‐genetic, opsin‐free neuromodulation.

Collectively, these emerging nanotechnologies illustrate capabilities that extend beyond the reach of conventional ophthalmic therapeutics, including molecular‐level programmability, spatiotemporal control of signaling, and responsiveness to physical or biochemical cues [155, 157, 158]. By enabling precise modulation of cellular pathways, gene regulation, immune interactions, and neuronal activity, such platforms address disease mechanisms that are difficult or impossible to access with traditional drugs or implants. Systematic investigation and adaptation of these advanced nanomaterials for ocular applications offer promising opportunities to expand the therapeutic landscape of ophthalmology and develop new strategies for treating retinal and optic nerve disorders.

## 5 Limitation and Future Outlook

While nanotechnology enables increasingly precise molecular targeting, its translation into ophthalmology remains constrained by significant knowledge and infrastructure gaps. Importantly, many nanotherapeutic strategies do not introduce entirely new therapeutic mechanisms, but instead enhance the delivery efficiency, stability, and tissue penetration of existing pharmacological agents through nanocarrier systems. In many cases, the therapeutic effect is therefore primarily derived from the encapsulated drug payload, with the nanomaterial functioning mainly as a delivery platform that improves bioavailability, prolongs intraocular retention, or enables controlled release. Recognizing this distinction is important when evaluating the clinical potential of nanomedicine approaches in ophthalmology [3, 17, 30, 33, 35].

A central challenge is the limited understanding of how nanoparticles interact with ocular tissues at both acute and chronic timescales [159]. The eye's distinctive barriers, including the corneal epithelium, inner and outer blood‐retinal interfaces, and the highly compartmentalized vitreous environment, require strict control of particle size, surface chemistry, and degradability to achieve therapeutic benefit without compromising tissue homeostasis [2, 3, 4, 5, 18, 160].

Biocompatibility and long‐term safety remain key concerns, particularly for metallic, carbon‐based, magnetoelectric, and optically responsive nanomaterials [18, 159, 160]. These systems may introduce risks such as photothermal tissue injury, oxidative by‐product accumulation, persistent microglial activation, or fibrosis formation, especially under repeated or prolonged dosing conditions [160, 161]. In addition, the ocular metabolic fate of many nanocarriers, including their biodegradation pathways, retinal biodistribution, and clearance through aqueous or lymphatic outflow, remains incompletely understood, highlighting the need for long‐term pharmacokinetic and toxicological studies in clinically relevant ocular models [48, 69, 70, 105, 106, 108, 115, 162, 163, 164, 165, 166]. Emerging nanosystems such as DNA origami, membrane‐coated particles, and magnetoelectric nanoparticles present additional uncertainties [150, 155, 157]. Their degradation pathways, intravitreal stability, off‐target effects, and clearance mechanisms have been explored mainly in non‐ocular models, leaving their relevance to retinal and anterior‐segment biology unresolved.

Translation is further challenged by manufacturing reproducibility. Many nanomaterials, especially stimuli‐responsive platforms and bioelectronic hybrids, exhibit physicochemical variability between batches. This complicates scalability, quality control, and compliance with Good Manufacturing Practice standards, particularly for systems requiring precise nanoscale architecture or multicomponent assembly [167, 168].

A related barrier involves regulatory ambiguity. Nanotherapeutics frequently exist at the interface between drugs, devices, and biologics, and current frameworks lack clear definitions for hybrid systems such as responsive implants, nanosensors, or bioelectronic nanointerfaces. As a result, clinical trial design often underutilizes molecular biomarkers that could better capture mechanisms such as neuroprotection, redox modulation, or angiogenic suppression [168].

Ethical and public‐acceptance considerations remain insufficiently addressed. Concerns related to long‐term residence of nanoparticles in ocular tissues, potential irreversibility of implanted nanosensors, or the integration of magnetoelectric or photothermal interfaces raise questions about permanence, autonomy, and risk. These issues highlight the need for transparent communication, accessible consent processes, and inclusive trial design to ensure responsible clinical adoption [169, 170].

With regard to the future outlook, emerging interdisciplinary approaches may help address several of these translational challenges. Advances in artificial intelligence (AI)‐driven nanomedicine design are beginning to accelerate the optimization of nanoparticle formulations by integrating materials science, pharmacokinetics, and biological response data [171, 172]. Machine‐learning frameworks can predict how nanoscale parameters such as particle size, surface charge, ligand density, and mechanical properties influence biodistribution, cellular uptake, and barrier penetration, potentially reducing the trial‐and‐error processes traditionally required for nanocarrier development [171, 172, 173]. In ophthalmology, where delivery efficiency is strongly constrained by anatomical barriers and small therapeutic windows, AI‐assisted design may help identify nanoparticle architectures capable of achieving optimal retinal penetration while minimizing off‐target exposure.

In parallel, digital and intelligent monitoring systems may enable a more precise evaluation of nanotherapeutic performance in vivo. Emerging approaches include nanosensors capable of detecting local biomarkers such as oxidative stress, inflammatory mediators, or intraocular pressure changes, as well as wearable or implantable devices that continuously monitor ocular physiology [174, 175, 176]. When combined with digital health platforms and data analytics, these technologies could support adaptive dosing strategies, early detection of adverse responses, and real‐time assessment of treatment efficacy [176, 177]. Such integration of nanotechnology with computational modelling, biosensing, and digital health systems may represent an important step toward more personalized and responsive ophthalmic therapies.

Together, these limitations underscore that while nanotechnology provides strong and exciting molecular tools for engaging ocular disease pathways, clinical translation requires parallel advances in mechanistic understanding, safety assessment, manufacturing reliability, and regulatory clarity. At the same time, emerging interdisciplinary approaches may provide new strategies to optimize nanotherapeutic development, improve safety evaluation, and support more adaptive and personalized ophthalmic treatment paradigms.

## 6 Conclusion

Nanotechnology is positioned to expand ophthalmic therapy because it can provide levels of control that conventional drugs and devices often cannot achieve. Across degenerative ocular diseases, treatment is limited by anatomical barriers, rapid clearance, and the need for sustained exposure in the posterior segment, while disease progression is driven by interacting pathways that include oxidative stress, inflammation, mitochondrial dysfunction, proteostatic failure, angiogenic signaling, and biomechanical remodeling. Engineered nanosystems can operate within biological interfaces that define early disease progression and can overcome the limited penetration, short retention, and insufficient pathway specificity associated with conventional drugs. Engineered nanosystems can improve tissue penetration and retention and enable controlled or stimuli‐responsive release, supporting a more durable and spatially targeted modulation of disease‐relevant mechanisms. In parallel, nanosensors and nano‐enabled imaging platforms may enable a closer linkage between molecular surveillance and therapeutic intervention. Diverse clinical and preclinical examples demonstrate the breadth of mechanistic regulation achievable with nanoscale materials. On the other hand, these advances also reveal persistent challenges that involve incomplete understanding of nanoparticle interactions with ocular tissue, uncertainties regarding chronic biocompatibility, variation in activation thresholds across microenvironments, and practical barriers related to manufacturing quality and regulatory definitions. Future progress will depend on integrating rigorous safety evaluation with mechanistic validation so that nanoscale precision can translate into durable molecular repair within ocular tissues. Within this trajectory, nanotechnology holds substantial potential to reshape ophthalmic care by aligning therapeutic intervention with the molecular origins of disease.

## Author Contributions

**Li Yao Jin:** writing – original draft, writing – review and editing. **Evashiniee G.P. Selvam:** writing – original draft, writing – review and editing. **Qinsong Mei:** writing – original draft, writing – review and editing. **Chi D. Luu:** writing – original draft, writing – review and editing. **Yeannie H.Y. Yap:** conceptualization, supervision, writing – original draft, writing – review and editing. **Daniel B.L. Teh:** conceptualization, funding acquisition, supervision, writing – original draft, writing – review and editing.

## Conflicts of Interest

The authors declare no conflicts of interest.

## Data Availability

Data sharing not applicable to this article as no datasets were generated or analyzed during the current study.
